# Avian Immunome DB: an example of a user-friendly interface for extracting genetic information

**DOI:** 10.1186/s12859-020-03764-3

**Published:** 2020-11-12

**Authors:** Ralf C. Mueller, Nicolai Mallig, Jacqueline Smith, Lél Eöery, Richard I. Kuo, Robert H. S. Kraus

**Affiliations:** 1 Department of Migration, Max Planck Institute of Animal Behavior, Am Obstberg, 78315 Radolfzell, Germany; 2 Department of Biology, University of Konstanz, Universitaetsstrasse 10, 78464 Konstanz, Germany; 3 HTWG Konstanz - University of Applied Sciences, Alfred-Wachtel-Str. 8, 78462 Konstanz, Germany; 4 The Roslin Institute and Royal (Dick) School of Veterinary Studies, University of Edinburgh, Easter Bush, Midlothian, Roslin, EH25 9RG UK

**Keywords:** B10K, Avian, Genomics, Immunogenomics, Immunology, Immunome, Trait database

## Abstract

**Background:**

Genomic and genetic studies often require a target list of genes before conducting any hypothesis testing or experimental verification. With the ever-growing number of sequenced genomes and a variety of different annotation strategies, comes the potential for ambiguous gene symbols, making it cumbersome to capture the “correct” set of genes. In this article, we present and describe the Avian Immunome DB (Avimm) for easy gene property extraction as exemplified by avian immune genes. The avian immune system is characterised by a cascade of complex biological processes underlaid by more than 1000 different genes. It is a vital trait to study particularly in birds considering that they are a significant driver in spreading zoonotic diseases. With the completion of phase II of the B10K (“Bird 10,000 Genomes”) consortium’s whole-genome sequencing effort, we have included 363 annotated bird genomes in addition to other publicly available bird genome data which serve as a valuable foundation for Avimm.

**Construction and content:**

A relational database with avian immune gene evidence from Gene Ontology, Ensembl, UniProt and the B10K consortium has been designed and set up. The foundation stone or the “seed” for the initial set of avian immune genes is based on the well-studied model organism chicken (*Gallus gallus*). Gene annotations, different transcript isoforms, nucleotide sequences and protein information, including amino acid sequences, are included. Ambiguous gene names (symbols) are resolved within the database and linked to their canonical gene symbol. Avimm is supplemented by a command-line interface and a web front-end to query the database.

**Utility and discussion:**

The internal mapping of unique gene symbol identifiers to canonical gene symbols allows for an ambiguous gene property search. The database is organised within core and feature tables, which makes it straightforward to extend for future purposes. The database design is ready to be applied to other taxa or biological processes. Currently, the database contains 1170 distinct avian immune genes with canonical gene symbols and 612 synonyms across 363 bird species. While the command-line interface readily integrates into bioinformatics pipelines, the intuitive web front-end with download functionality offers sophisticated search functionalities and tracks the origin for each record. Avimm is publicly accessible at https://avimm.ab.mpg.de.

## Background

Ever since the advent of commercial next-generation sequencing platforms in the early 2000s with its associated decrease in sequencing costs [1], the number of DNA sequences increased considerably [2]. Generally, these data become publicly accessible in databases provided by projects focussing on different aspects of biological sequence information [3, 4]. Ensembl [5] and NCBI [6] for instance, have a strong focus on genome annotation with the help of RNA transcript information while UniProt has a pronounced emphasis on protein-coding genes and biological function of proteins. UniProt’s records are either based on manually annotated, non-redundant protein sequences (SwissProt) or on high-quality computationally analysed records, which are enriched with automatic annotation (TrEMBL) [7]. Relying on accurate genome annotations and protein descriptions, Gene Ontology (GO) [8, 9] categorises gene products and fits them into a computational model of biological systems. Their assignment deploys a controlled vocabulary, so-called GO terms, to link genes and gene products to biological processes, cellular components, or molecular functions.

However, genome annotation is not standardised, and each service provider uses their own custom-built annotation pipelines. As a consequence, this often leads to ambiguity in gene names during genome annotation with different gene symbols being given to the same gene or the same gene symbol being given to different, but similar genes. Additionally, since the pre-existing wealth of sequencing information relies on model organisms like human and mouse, there is a strong bias in gene symbols towards those chosen for these species. Particularly for model species, this issue has been partially addressed, for example by the Human Genome Organisation (HUGO) Gene Nomenclature Committee (HGNC) [10], the Vertebrate Gene Nomenclature Committee (VGNC) [11], or the Chicken Gene Nomenclature Consortium [12]. However, this neither guarantees that gene names are harmonised among these consortia, nor does it keep researchers from assigning alternative gene symbols in their annotations, especially when working with non-model species. All these constraints during the data acquisition step can be very challenging to overcome while preparing a gene-related study where choosing the “correct” set of genes is a fundamental and repetitive task for each gene [13]. In other words: “Now that next-generation sequencing (NGS) is commonplace in many laboratories and that efficient bioinformatics toolkits have been developed, knowledge extraction is the bottleneck of genomics” [14].

Gene Ontology’s web-based tool AmiGO 2 [15] offers a convenient entry point for searching and browsing genes and gene products. These are hierarchically organised following the top three categories “biological process”, “cellular component” and “molecular function”. For instance, researchers addressing vision can expand to “biological process/behaviour/visual behaviour” and select genes in this category with the option to further narrow down the results to a focal taxon. Olfaction-related genes can be found under “molecular function/binding/odorant binding”, or vocal learning-related genes under “biological process/behaviour/vocalisation behaviour”. The information for each entry includes the GO term, the source of evidence, and a gene symbol. The gene symbol can then be looked up for further information, such as the nucleotide sequence or the amino acid sequence in Ensembl or UniProt, respectively. This information can be used to prepare gene expression experiments, to conduct gene evolution studies, to look into functional variation, to compare genes between different taxa, or to prioritise targets for gene-editing technologies. These repetitive preparatory steps, however, become the more cumbersome, the more genes are considered.

The immune system in vertebrates is an example of a highly complex biological process [16, 17] represented by more than 25,000 gene products, according to the category “immune system process” in Gene Ontology (filtered for Vertebrata, GO release 2020-06-01). Within the scope of medical research, many immune-related studies have been conducted in human and mouse.

The avian immune system is quite different to that of mammals and is exhibited by different immune organs, cell types and gene repertoires. A fundamental difference between mammals and birds is the absence of lymph nodes but the presence of diffuse lymphoid tissue in birds [18]. As they lack lymph nodes, chickens are thus also missing the genes for the lymphotoxins and lymphotoxin receptors. The lack of functional eosinophils correlates with the absence of the eotaxin genes and the previously reported observation that interleukin-5 (IL-5) is a pseudogene [19].

Other structural and functional differences between the immune systems of birds and mammals include the architecture of the MHC [20] and different modes of somatic recombination in the generation of antibody diversity. Humans have a set of IgM, IgD, IgG, IgA, and IgE antibodies, whereas chickens have IgA, IgM and IgY (this being the equivalent of mammalian IgG).

Some immune genes are either completely missing in birds or are only present in particular species [21] and immune gene families have different orthologues between species, for example, the numbers and types of cytokines and chemokines [22]. It is therefore important that we have a means of cataloguing and a tool for searching these essential genes in avian species.

Immune system-related research in birds is crucial to better understand, for instance, the spread of zoonotic diseases which is not only important in the context of species conservation but also has a potentially great impact on human health and economy (livestock). Birds are highly mobile with some migrating long distances and are thus a potential reservoir and vector for zoonotic diseases [23–27]. Therefore, eco-immunology [28, 29] has gained momentum over the last decade [21, 30, 31].

Recently, the Bird 10,000 Genomes (B10K) Project [32, 33] completed its second phase where the genome of at least one bird species per family was sequenced [Feng et al., *in review*]. This resulted in 363 bird genomes sequenced on Illumina platforms. We aggregated the immune genes of these genomes to make them publicly and easily accessible in the Avian Immunome DB (Avimm) at https://avimm.ab.mpg.de [34]. This data was complemented with transcript information and amino acid sequences available in Ensembl and UniProt, respectively.

Within the scope of this project, we define the immunome as the set of genes that fall into the category “biological process/immune system process” according to Gene Ontology. However, we are aware of the difficulty of defining what an immune gene is and what is not [35]. Furthermore, we consider our generalist database to be complementary or an entry point to specialist databases such as the international ImMunoGeneTics information system^®^ (IMGT^®^, [36]) and acknowledge that we rely on immune genes with associated gene symbols that are available on Gene Ontology, Ensembl, or UniProt.

The only model species in birds where the whole immune system has been studied intensively and for a long time is the chicken (*Gallus gallus*), mostly for agricultural reasons [37]. The chicken immune system is arguably most comprehensively described with 1488 hits in Gene Ontology (GO release 2020-06-01) as compared to other well-studied bird species (five hits in mallard (*Anas platyrhynchos*), and three hits each in the zebra finch (*Taeniopygia guttata*) and collared flycatcher (*Ficedula albicollis*)). This project aims to compile available information about the avian immunome and at the same time disentangle gene symbol ambiguities by linking genes with different names (symbols) to the same unique gene identifier (gene_id in the database). As a consequence, all alternative gene symbols that are found on Ensembl and UniProt are included. Since there is no general standardisation or harmonisation of gene symbols, this intentionally inclusive approach allows for searching our database using different names for the same gene and thus, facilitates comparative studies. Canonical gene symbols (based on Ensembl) are highlighted, and original identifiers of each record are retained and linked to the source provider.

**Fig. 1:**
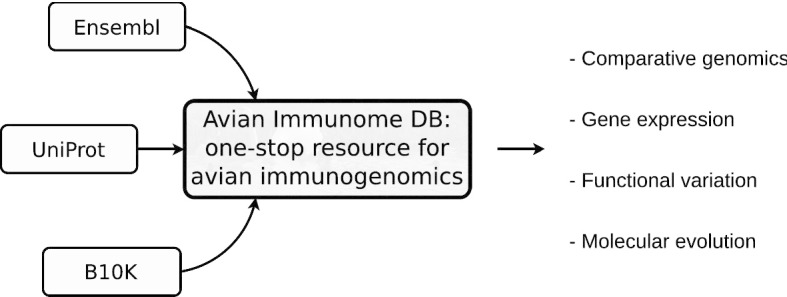
Concept of Avimm, the Avian Immunome DB

Currently, the database contains evidence for avian immune genes from Ensembl, UniProt and B10K. Avimm strives to become a one-stop resource for avian immunogenomics to facilitate studies in comparative genomics, studies looking into functional variation and the molecular evolution of immune genes and their pathways on whole-genome level [38], as well as an entry point for immune gene expression experiments in birds [39] (Fig. 1). Avimm can be searched by gene symbols and species through the web interface or query script, and the results (lists, annotations, nucleotide and amino acid sequences) can be readily downloaded in various formats.

Furthermore, Avimm is a case study to demonstrate the utility of our database layout and implementation for biological processes other than the immune system, for example, olfaction or vocal learning. At the same time, the database concept can be applied to virtually any taxonomic group or rank. An extensive description of the database design is available on our project’s repository [40], which provides scripts and instructions to facilitate the setup of similar databases.

## Construction and content

**Fig. 2:**
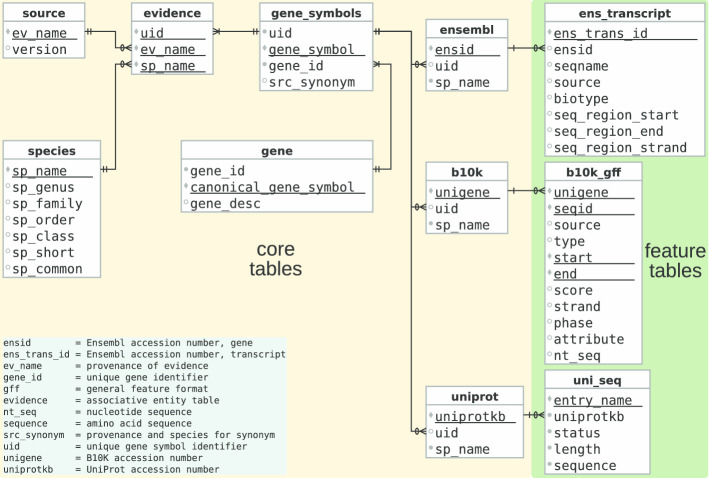
Logical data model (LDM) in Crow’s Foot Notation [42] of Avimm. Core tables represent the basic structure of the database and link the evidence-specific identifiers to Avimm’s unique identifiers (uid). Feature tables represent evidence-specific features like isoforms, nucleotide sequences, or amino acid sequences of the immune genes

A detailed reconstruction of Avimm is described in our wiki [41]. In brief, the relational database MariaDB (v10.1.44) was installed on a Linux server (Ubuntu 18.04.4 LTS) and secured according to MariaDB’s documentation. The logical data model (LDM) consists of core tables, feature tables and their relationship to each other. The core tables keep track of essential information like species and taxonomy, the origin of evidence and their release cycle versions, gene symbols and descriptions, original evidence identifiers (accession numbers), and resolution of many-to-many relationships (mapping of identifiers). The feature tables contain transcript information, including isoforms, annotation, and nucleotide and amino acid sequences (Fig. 2).

### Data acquisition and import

As a starting point, genes based on “biological process/immune system process” were selected in AmiGO 2 and further filtered for the best-studied model bird chicken (*Gallus gallus*, GO release 2020-06-01). These gene symbols were used as a “seed” for creating Avimm. First, the core tables were loaded, including gene synonyms. Feature tables were then subsequently loaded. Detailed scripts and descriptions are available on Avimm’s GitLab repository [40].

By using the gene symbols obtained from GO, Ensembl (release 100, Apr 2020) was queried by a Python script using the Ensembl public MySQL servers [43]. The script queries Ensembl’s REST interface [44] for available species and their current releases [45]. After that, the script checks which of the species are contained in B10K’s current annotation of 363 bird species. For each of these species, the script connects to the corresponding Ensembl database and downloads all Ensembl stable IDs (Ensids) associated with a gene symbol contained in the “seed”. In this process, also the synonyms are checked (table external_synonym in Ensembl). If a gene symbol of the “seed” is found in the synonyms, the corresponding Ensid is downloaded as well. The Ensids were then loaded into Avimm. In the next step, all gene synonyms for the Ensids loaded into Avimm were downloaded from Ensembl. Another Python script was used which connects to each of the relevant species databases and downloads the synonyms for each Ensid contained in Avimm. These synonyms were then also loaded into Avimm.

UniProt data were obtained from the UniProt website. For this purpose, a list of Ensids contained in Avimm was prepared. With this list, the UniProt web interface [46] was queried, and the resulting data were downloaded filtered by the following columns: “Entry (ID)”, “Entry name”, “Status (reviewed/unreviewed)”, “Protein names”, “Organism”, “Gene names (primary)”, “Gene names (synonym)”, “Length”, “Sequence”, “Your list”. As an initial step, only Entry IDs (UniProtKBs) were loaded into Avimm.

For the import of the B10K data, all gene symbols found in Avimm (including synonyms) were used. For each of these symbols, it was checked whether the gene symbol was found in the B10K genomes’ annotations. If the gene symbol was found, the B10K ID (Unigene; B10K-internal identifier and not to be confused with NCBI’s Unigene) was extracted and imported into Avimm.

**Fig. 3:**
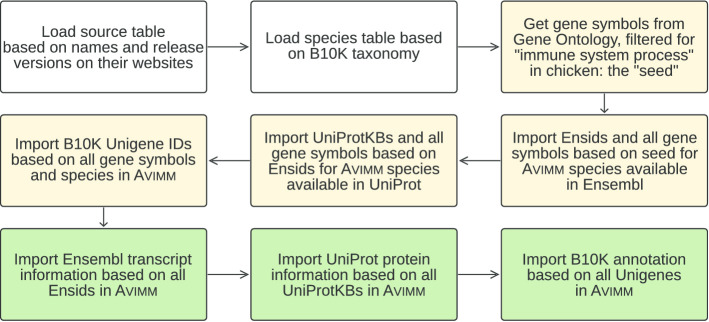
Flow chart, loading of Avimm. Uncoloured boxes show preparatory steps. Yellow boxes describe imports into core tables and green boxes imports into feature tables. The initial set of immune genes in chicken is derived from “biological process/immune system process” in AmiGo 2 filtered for chicken. Gene symbols were extracted from Ensembl’s website since Ensembl has the most current/complete chicken annotation. All steps are explained in detail on the project’s wiki page [41]

To fill the feature tables, all obtained accession numbers from Ensembl, UniProt and B10K were then used to extract transcript information, amino acid sequences and nucleotide sequences, respectively, which were subsequently imported into Avimm (Fig. 3). Data were imported via a stored procedure (function in the database) which performs consistency checks before importing data.

Synonyms of gene symbols were handled in the following way: The primary gene symbol used in Ensembl was stored as a canonical name in Avimm and identified by a unique gene identifier (gene_id). The synonyms found in Ensembl were also imported into Avimm. Each gene symbol (primary or synonym) received an additional unique gene symbol identifier (uid). This uid was used to identify the gene symbol when storing and extracting data. Synonyms were linked to their canonical name via gene_id. UniProt was queried using the Ensembl stable IDs (Ensids) stored in Avimm. Additional gene symbols found by this process were also stored in Avimm as synonyms with a uid and linked to the canonical name via gene_id. As such, uid uniquely identifies a gene symbol while gene_id identifies a gene. The link between uid and gene_id allows for finding all data related to a gene. For a user it is sufficient to know one of the gene symbols corresponding to a gene (either the canonical name or a synonym); the link between uid and gene_id allows to identify the canonical gene name.

### Gene properties in the database can be accessed in two ways

A Python script qimm.py (QueryIMMunome) was written as a command-line interface (CLI) to query data and output nucleotide and amino acid sequences in fasta format [40]. This script can be readily incorporated in bioinformatics pipelines. As a graphical user interface, a web front-end was developed, which allows for easy access to the data in Avimm [34], independent of specialist bioinformatics skills.

Django [47], a widely used web framework built on Python that is known to facilitate the development of web applications, was used for the development of Avimm’s web interface. With both the CLI and the web interface written in Python, it was effortless to reuse the program logic written for the CLI in the web interface. The web interface has two primary purposes: collecting the parameters needed by qimm.py in a user-friendly way and presenting the query results. The parameters collected by the web interface are fed into qimm.py, which queries the database and returns the results, which are then displayed by the web front-end.

**Fig. 4:**
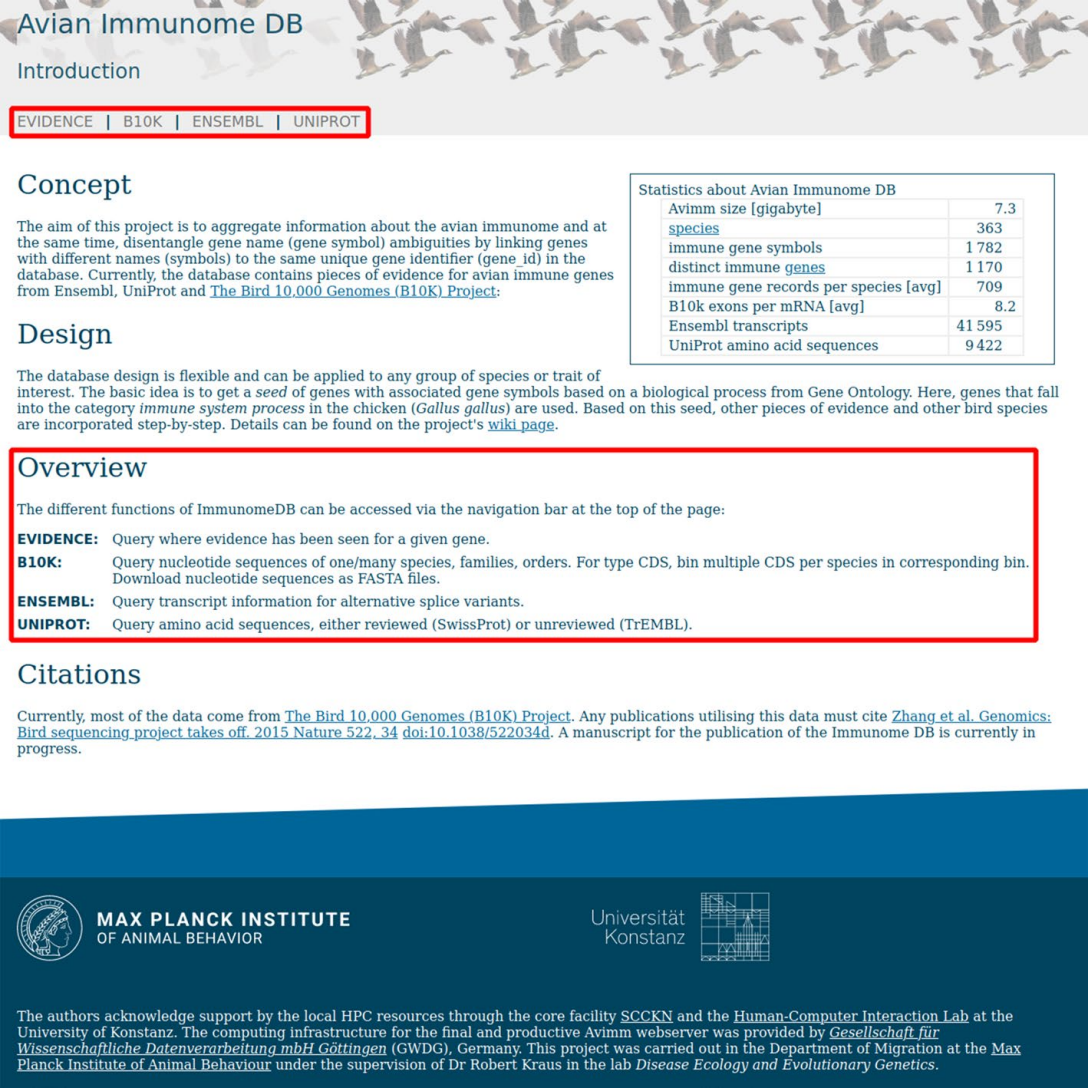
Avimm landing page with links to the four function pages (upper box) and description for each function (lower box)

The web front-end consists of the main page with a general description of the project and four function pages (Fig. 4), each one corresponding to one of the main modules/functions provided by qimm.py. These function pages provide user-friendly methods to enter query parameters.

**Fig. 5:**
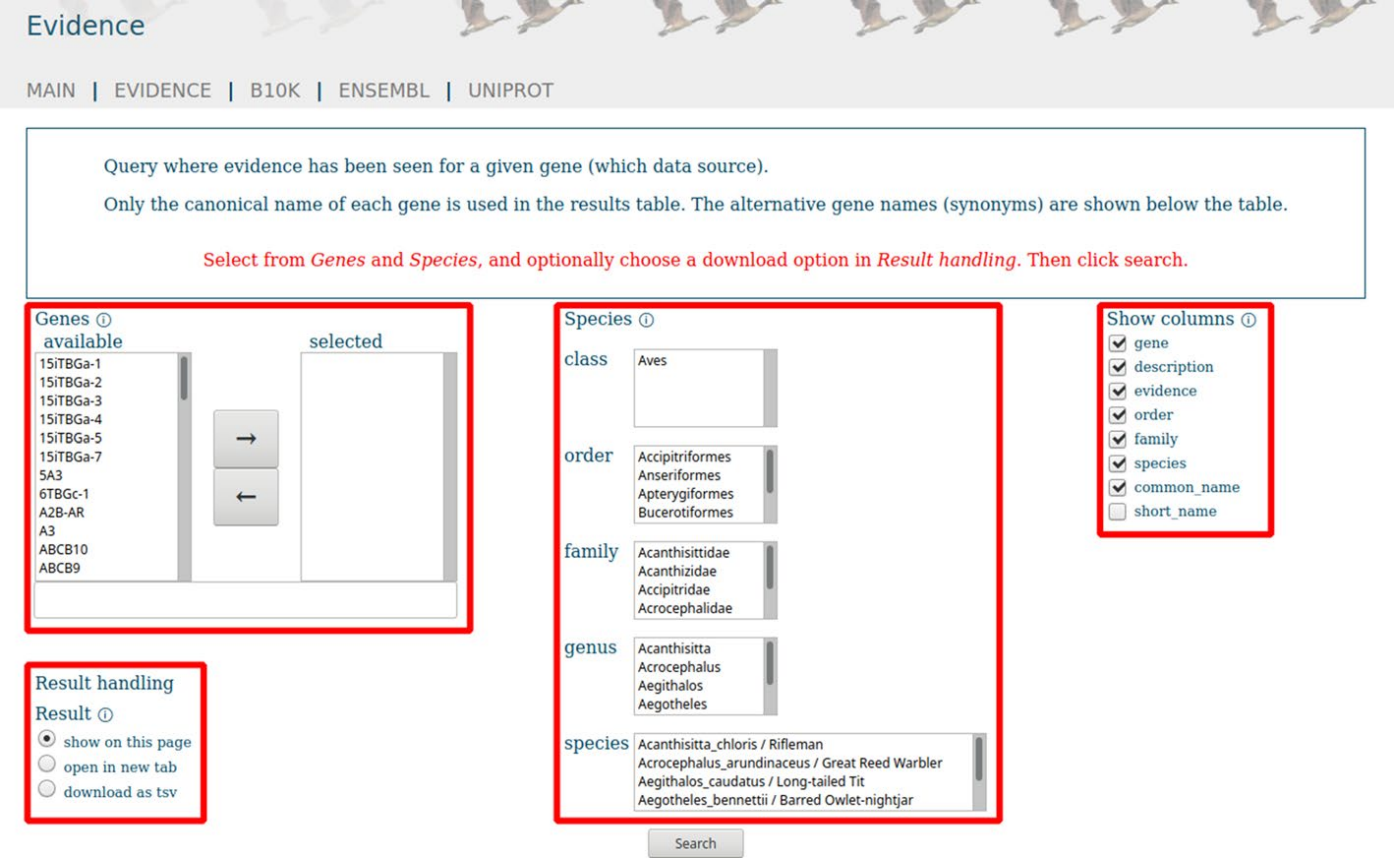
Excerpt of evidence function page. Gene selection either by “mark-and-arrow” or free text (top left box). Species selection based on taxonomic ranks (centre box). Checkboxes to filter columns (right box) and download data option (bottom left box). Each section has an information button (circled “i”) to provide further details

For example, gene symbols are presented in two lists: one containing all available gene symbols, the other the selected gene symbols. Marked symbols can be moved from one list to the other. Besides this, gene symbols can be entered into the text field on the bottom, for example, by copy and paste. Species can be selected by a set of lists representing the taxonomic ranks starting at the class level. Once a selection in an upper level is made, the lists in the lower levels only contain the values compatible with the selection in the upper level. Which data to be displayed can be selected via checkboxes. For other options, for example, downloading data, checkboxes are also provided (Fig. 5).

The web front-end has the additional benefit that hyperlinks can be used. As a consequence, the result page of the evidence function provides direct links to the function pages of Ensembl, UniProt and B10K with the same selection of gene symbols and species that have been used on the evidence function page. Accession numbers in the results of Ensembl and UniProt queries are linked to the corresponding entries within Ensembl and UniProt, respectively. The B10K function page provides links to NCBI BLAST [48] for each result record. The Ensembl function page provides links for each record to the corresponding gene in Ensembl and NCBI. The UniProt function page provides links to the corresponding protein in UniProt and NCBI’s protein blast (BLASTP).

## Utility and discussion

The current size of Avimm is 7.3 GB, which encompasses 363 bird species with an average number of 709 immune genes per species [Table 1]. Within the 1782 immune gene records, 363 genes with more than one distinct gene symbol were identified. The gene IL8 (Interleukin-8), for example, is pointing to nine synonymous gene symbols (CEF4, CXCL13, CXCL13L2, CXCL8, EMF1, gIL-8GN, IL8L1, interleukin-8 and k60).

**Table 1:** Statistics of the Avian Immunome DB

Avimm size [gigabyte]	7.3
Species	363
Immune gene symbols	1782
Distinct immune genes	1170
Immune gene records per species [avg]	709
B10K exons per mRNA [avg]	8.3
Ensembl transcripts	41,595
UniProt amino acid sequences	9422

### Examples from the CLI

A quick overview of what evidence is available in the database is given below, using the gene IFNL3A (Interferon lambda-3 A) across all species and can be accomplished on the command line:
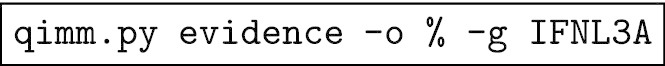


Explanation of arguments:

the script’s name
query the evidence table
all orders (SQL wild card % for all entries)
the gene of interest

This query will return a species list for which there is evidence for this gene and the type of evidence (B10K, Ensembl or UniProt). Names of alternative gene symbols will be printed at the bottom (in this example, IFNL, IFNL3, IL-28B, IL28 and IL28B are synonyms for IFNL3A). Note that the same results will be returned when querying, for instance, with “-g IL28” since it maps to the same gene in Avimm.

The Ensembl tables need to be queried to retrieve transcript information:
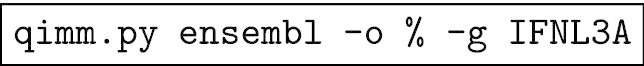


This query will return the Ensembl accession numbers for the genes and transcripts which can then be pasted in Ensembl’s website [49] to get further information.

Amino acid sequences for the gene product are queried through UniProt:
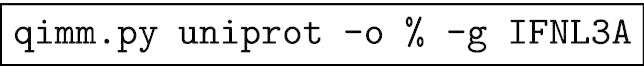


This query will return a fasta-formatted output of the amino acid sequences and adding the argument “-w” to the query will generate fasta output files which can then be used as input for an alignment software for further downstream analyses of these sequences.

Nucleotide sequences of the mRNA can be queried using the B10K module:
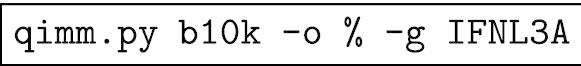


The output is again fasta-formatted and adding “-w” will generate fasta files which can be used for downstream analyses of these nucleotide sequences of IFNL3A. A more detailed list of examples, including generated output, can be found on Avimm’s GitLab repository [40].

For instance, a simple gene presence-absence analysis of the gene MASP2 in Avimm,
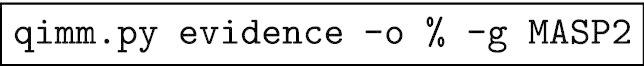


shows evidence for almost all Anseriformes (5/7) and all Galliformes (11/11), but only for two Passeriformes (2/173) in the database. In humans, MASP2, mannan-binding lectin-associated serine protease-2, is involved in the activation of the lectin pathway of the complement system. MASP2 deficiency was associated with increased susceptibility to infections or autoimmune diseases [50]. The order-biased evidence immediately raises two questions: Is MASP2 mostly absent in Passeriformes, or is it a sequencing (or annotation) artefact? If the former case was true, why is it present in most Galloanserae?

### Examples of web front-end

**Fig. 6:**
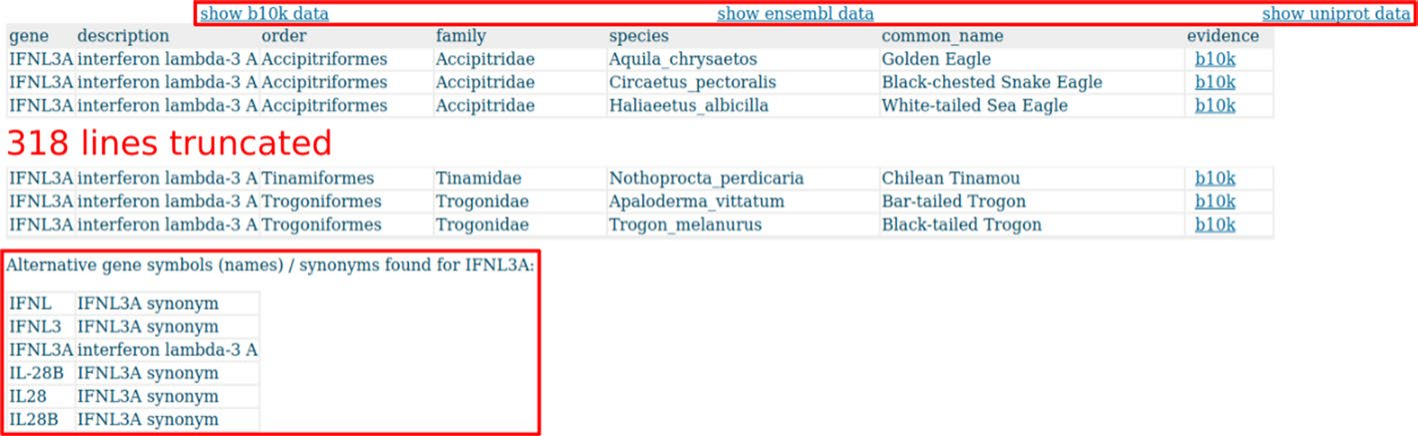
The gene evidence result list shows one record for each evidence of the gene IFNL3A in the selected species. The columns can be sorted (ascending or descending) by clicking on the corresponding column header. On top of the list are links to B10K, Ensembl, and UniProt result pages based on the same search criteria (upper box). If there are alternative gene symbols for the query gene in Avimm, then these are listed on the bottom of the page (lower box)

Evidence for the gene IFNL3A (Interferon lambda-3 A) across all species in Avimm can also be found via web front-end [51]. On top of the result list, there are direct links to B10K, Ensembl, and UniProt result pages based on the same search criteria. On the bottom of the page, alternative gene symbols are listed (Fig. 6).

**Fig. 7:**
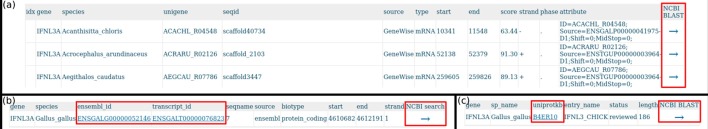
Excerpts of the result pages for IFNL3A in B10K (**a**), Ensembl (**b**), and UniProt (**c**). Each result page offers additional filters and download options. The original identifiers of the source of evidence are retained and linked to the original entries on Ensembl (**b**, left box) or UniProt (**c**, left box). Additionally, links to NCBI nucleotide sequence BLAST (**a**, box), NCBI gene information (**b**, right box), and NCBI amino acid sequence BLAST (**c**, right box) are provided for each record

The B10K link leads to the B10K results page with annotation and further options (filters, data download) including a link to NCBI’s nucleotide BLAST page for each record (Fig. 7a). The Ensembl link leads to Ensembl transcript information, including links to Ensembl’s gene and transcript IDs, and a link to NCBI’s gene search page for each record (Fig. 7b). The UniProt link leads to UniProt protein information and further options (filters, data download) including a link to UniProt’s ID, and a link to NCBI’s protein BLAST for each record (Fig. 7c).

**Fig. 8:**
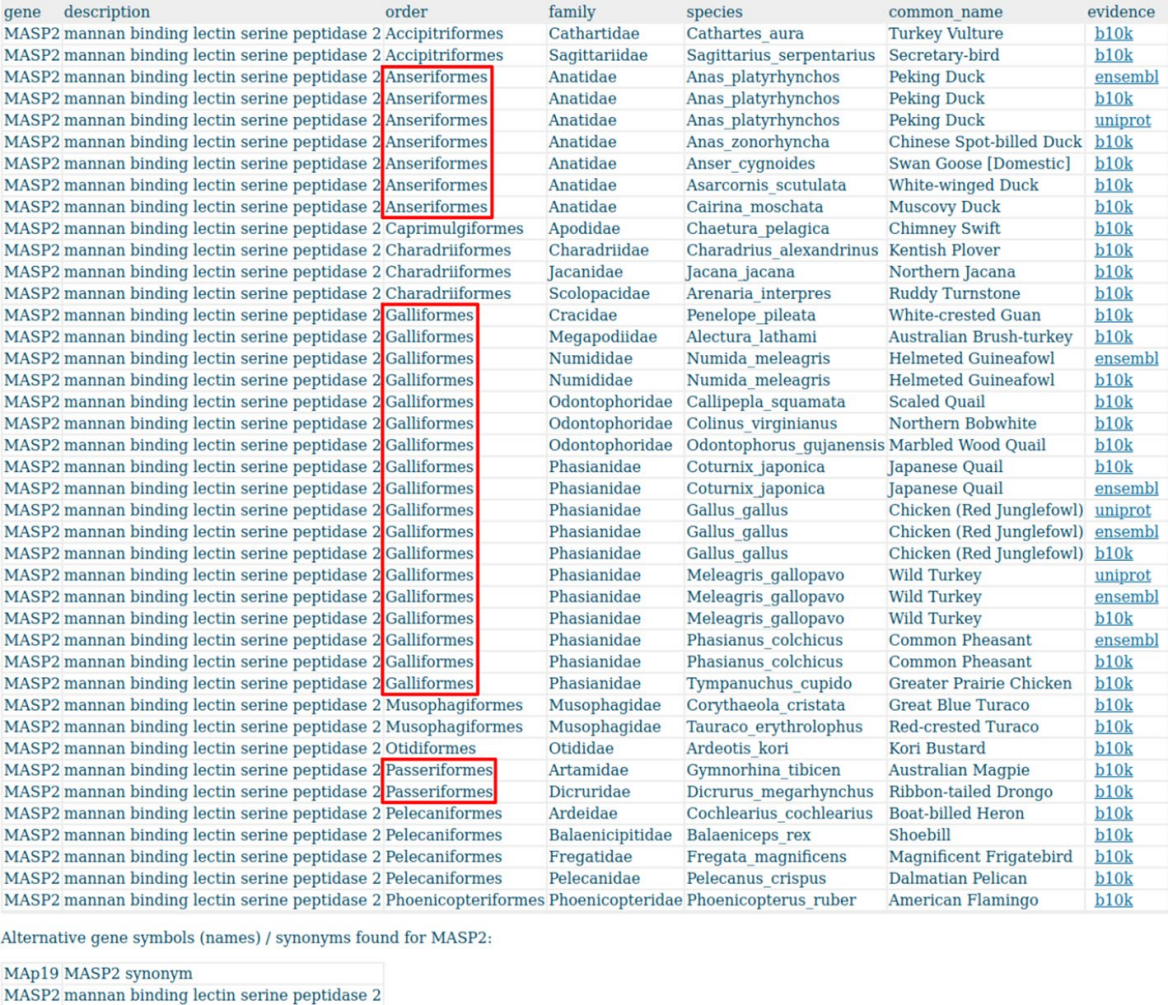
Excerpt of the result page for MASP2 evidence across all 363 species in Avimm. There is evidence in all Galliformes (11/11) and almost all Anseriformes (5/7) but only in two Passeriformes (out of 173 in Avimm)

Absence-presence analysis of the gene MASP2 across all 363 species in Avimm shows evidence in almost all Galloanserae (16/18) whereas there is evidence in only two (out of 173) Passeriformes (Fig. 8).

### Scope and context

Avimm is a representation of the currently available information regarding avian immune genes and can potentially economise repetitive, preparatory steps for studies in comparative genomics, gene expression, functional variation and molecular evolution of immune genes. This is ensured by a pre-selection of immune genes based on the well-annotated chicken genome as well as an automated resolution, incorporation and representation of ambiguous gene symbols. A quick presence-absence analysis of immune genes for available species in Avimm will become even more useful with the next data release of the B10K consortium (phase III; at least one representative bird genome per genus covering 2,500 genera and subgenera) [32]. The sevenfold increase of genomes and annotations will also mitigate the current limitation of containing only one or a few bird species representatives per bird family. For each entry, original identifiers (accession numbers of Ensembl, UniProt and B10K) are retained which facilitates the gathering of additional gene information from these providers. The accompanying query script qimm.py can be readily incorporated into bioinformatics pipelines to analyse nucleotide and amino acid sequences in fasta format, for instance, for alignments and SNP identification. The database is already prepared to incorporate immune genes of other vertebrate animal classes to further extend the capability for comparative genomics. For this, a stored procedure takes care of necessary consistency checks before importing new data, which will also be an advantage for the incorporation of immune genes of the forthcoming hundreds of platinum-quality genomes from the Vertebrate Genomes Project [52]. These genomes of birds and other vertebrate classes will be of much higher sequencing quality and read contiguity, which will presumably uncover previously undetected immune genes [53, 54]. Connected to this, particularly in the current version, one should keep in mind that the absence of a particular gene in a species does not necessarily reflect biological reality but might be the consequence of the shortcomings of short-read sequencing [54].

Our generalist database is designed in a way to harbour the common denominator of a focal trait and taxonomic group, mainly a broad range of non-model organisms. It relies on gene symbols found in established projects like Ensembl or UniProt. Specialist databases include in-depth expertise on focal genes and gene products for a given species. For example, IMGT [36] has a strong focus mainly on immunoglobulins and T cell receptors in vertebrates. They also assign gene symbols which then have to be approved by HGNC [10] and VGNC [11] before they are eventually incorporated to other generalist databases. Therefore, we recommend to also consult specialist databases after a pre-selection of focal genes in Avimm. Eventually, genes and gene products with assigned gene symbols will be included within Ensembl and UniProt, and will thus also be included in Avimm.

With the release cycles of Ensembl (every three months) and UniProt (every month) it is challenging to keep up-to-date with the most current annotations; all the more, considering that the number of annotated highly contiguous whole-genomes is ever-growing (five bird genomes in Ensembl release 95 but already 40 in release 100) and the automated annotation pipelines of these service providers rapidly increase the number of correct immune gene annotations. Additional Python scripts are provided on the project’s GitLab repository to facilitate the incorporation of new evidence into Avimm.

An essential feature of the relational database design is the internal mapping of genes and identifiers to unique identifiers through uids and gene_ids. This allows the resolution of many-to-many relationships and, in consequence, is the foundation for allowing queries based on any well-known gene symbol for a single gene.

## Conclusions

Currently, Avimm proves to be a valuable resource with which to extract avian immune gene information, mainly from the B10K phase II annotation. With phase III, a total of circa 2500 genomes spanning all bird genera are expected [32]. Although only limited transcript information and amino acid sequences are currently available, Ensembl and UniProt continually annotate genomes that are uploaded to their repositories. These annotations can then be easily added to Avimm. Additionally, VGP has completed or nearly completed 38 of 95 proposed bird genomes of the highest sequencing and assembly quality currently available. These genomes will be annotated, and extracted immune gene information can also be added to the database. It is currently advisable to re-create affected tables in Avimm which is facilitated by Python scripts on the project’s website to reflect the release cycles of Ensembl and UniProt.

The organisation of the data in a relational database and the separation of core and feature tables makes it easy to add additional sources and properties of avian immune genes. For the same reasons, it is also possible to apply the database design to other biological processes such as olfaction, vision or vocal learning. Specialist communities in comparative and functional genomics in their respective disciplines face similar or even identical challenges with genetic information scattered across research consortia and databases, with quite a few gene symbols showing ambiguity. Furthermore, the database design is not limited to birds but can be used to represent genomic data for other taxa with little effort. It is possible to then link databases for different taxa or properties. We provide information on how to use our database model for other taxa or other sets of genes in our wiki. Finally, through the intuitive web interface, data acquisition from international consortia and databases becomes less cumbersome for non-bioinformaticians, who otherwise would need to consult multiple data sources with frequently ambiguous gene symbols.

## Data Availability

https://gitlab.com/rcmueller/immunomedb, https://avimm.ab.mpg.de.

## Abbreviations

Avimm: Avian Immunome DB
B10K: Bird 10,000
BLAST: Basic local alignment search tool
CDS: Coding sequence
CLI: Command-line interface
DB: Database
Ensid: Ensembl stable ID (accession number)
GB: Gigabyte
GO: Gene ontology
LDM: Logical data model
MHC: Major histocompatibility complex
mRNA: Messenger RNA
NCBI: National Center for Biotechnology Information
NGS: Next generation sequencing
REST: Representational state transfer
RNA: Ribonucleic acid
SNP: Single-nucleotide polymorphism
SQL: Structured query language
uid: Unique (gene symbol) identifier
Unigene: B10K accession number (not to be confused with Unigene Laboratories)
UniProt: Universal Protein database
UniProtKB: UniProt Knowledgebase (accession number)
VGP: Vertebrate Genomes Project
