# Correction to: Avian Immunome DB: an example of a user‑friendly interface for extracting genetic information

**DOI:** 10.1186/s12859-021-04388-x

**Published:** 2021-09-30

**Authors:** Ralf C. Mueller, Nicolai Mallig, Jacqueline Smith, Lél Eöry, Richard I. Kuo, Robert H. S. Kraus

**Affiliations:** 1 Department of Migration, Max Planck Institute of Animal Behavior, Am Obstberg, 78315 Radolfzell, Germany; 2 Department of Biology, University of Konstanz, Universitaetsstrasse 10, 78464 Konstanz, Germany; 3 HTWG Konstanz - University of Applied Sciences, Alfred-Wachtel-Str. 8, 78462 Konstanz, Germany; 4 The Roslin Institute and Royal (Dick) School of Veterinary Studies, University of Edinburgh, Easter Bush, Midlothian, Roslin, EH25 9RG UK

## Correction to: BMC Bioinformatics (2020) 21:502 10.1186/s12859-020-03764-3

Following publication of the original article [1], the authors identified an error in the author name of Lél Eöry.

The incorrect author name is: Lél Eöery.

The correct author name is: Lél Eöry.

The author group has been updated above and the original article [1] has been corrected.
